# Artificial Intelligence for Image Analysis in Oral Squamous Cell Carcinoma: A Review

**DOI:** 10.3390/diagnostics13142416

**Published:** 2023-07-20

**Authors:** Vanesa Pereira-Prado, Felipe Martins-Silveira, Estafanía Sicco, Jimena Hochmann, Mario Alberto Isiordia-Espinoza, Rogelio González González, Deepak Pandiar, Ronell Bologna-Molina

**Affiliations:** 1 Molecular Pathology Area, School of Dentistry, Universidad de la República, Montevideo 11400, Uruguay; vanesapereira@odon.edu.uy (V.P.-P.); felipemartins@odon.edu.uy (F.M.-S.); estefania.sicco@pedeciba.edu.uy (E.S.); jhochmann@fcien.edu.uy (J.H.); 2 Department of Clinics, Los Altos University Center, Institute of Research in Medical Sciences, University of Guadalajara, Guadalajara 44100, Mexico; mario.isiordia@academicos.udg.mx; 3 Research Department, School of Dentistry, Universidad Juárez del Estado de Durango, Durango 34000, Mexico; rogelio.gonzalez@ujed.mx; 4 Department of Oral Pathology and Microbiology, Saveetha Dental College and Hospitals, Chennai 600077, India; deepakp.sdc@saveetha.com

**Keywords:** artificial intelligence, deep learning, digital image, histopathological analysis, machine learning, oral squamous cell carcinoma

## Abstract

Head and neck tumor differential diagnosis and prognosis have always been a challenge for oral pathologists due to their similarities and complexity. Artificial intelligence novel applications can function as an auxiliary tool for the objective interpretation of histomorphological digital slides. In this review, we present digital histopathological image analysis applications in oral squamous cell carcinoma. A literature search was performed in PubMed MEDLINE with the following keywords: “artificial intelligence” OR “deep learning” OR “machine learning” AND “oral squamous cell carcinoma”. Artificial intelligence has proven to be a helpful tool in histopathological image analysis of tumors and other lesions, even though it is necessary to continue researching in this area, mainly for clinical validation.

## 1. Introduction

Oral cancer is the sixth most common cancer around the world, with a high risk of morbidity and mortality [1]. Its diagnosis largely relies upon the correlation of clinical features and histopathological parameters, with more than 90% of the cases being morphologically diagnosed as oral squamous cell carcinoma (OSCC) [2]. OSCC is an aggressive type of oral cancer, and its prognosis can be affected according to diagnostic delay [3]. The diagnosis of OSCC is sometimes difficult to establish, mainly due to the heterogeneity of the clinical lesions associated with the subjectivity that the histopathological interpretation of some cases may present, according to the empirical experience of the oral pathologist community [4].

Further, given the existing controversy in the differential diagnosis of various head and neck cancers, achieving an objective histomorphological characterization based on novel technologies arises as an auxiliary tool for its interpretation with digital images. In recent decades, important advances have been made in the field of microscopy as a result of scientific and technological development in four fundamental aspects: optical parameters (improving contrast, spatial resolution, and reducing aberrations), sensors (compact digital cameras with sensors of high resolution, allowing increasingly better quality images at lower costs), the lighting sources that are used (which are more stable, precise and economical light sources with less environmental impact), and the computational network (both in hardware and in software, allowing to acquire, store, process, analyze and even reconstruct images of greater complexity and quality) [5]. At present, histopathological analysis of tissue biopsies by an oral pathologist is the gold standard for the diagnosis of OSCC. Slide digital scanners have provided new insight into tissue histopathology with several advantages in the field, such as making possible the application of computerized image analysis and machine learning (ML) techniques. Algorithms are now being developed for research, disease detection, diagnosis, and prognosis prediction, supporting the view of the pathologist. This objective characterization and pattern recognition of tissues and structures in digital slides is important not only from a diagnostic point of view but also in order to understand the biological mechanisms of the pathological process for research purposes. In this review, we briefly focused on the novel applications of digital histopathological image analysis in OSCC.

## 2. Materials and Methods

A literature search was performed in PubMed—MEDLINE online database in order to develop the present review. The keywords that were used in the portal were the following: “artificial intelligence”, “deep learning”, “machine learning”, and “oral squamous cell carcinoma”. These words were combined with the booleans “AND” and “OR” in the following order: “artificial intelligence” OR “deep learning” OR “machine learning” AND “oral squamous cell carcinoma”. The literature search was carried out from inception until May 2023 without any date restrictions applied. Moreover, a manual search was performed in the references of the selected articles in order to identify additional studies. Additionally, restricted access articles were recovered from the institutional access of Universidad de la República called “TIMBO”.

The digital search results yielded a total of 419 articles. The titles and abstracts of each article were analyzed, and the articles containing relevant information were selected for full-text evaluation. Finally, a total of 42 articles were selected and included in the present review.

## 3. General Concepts of Artificial Intelligence

**Figure 1:**
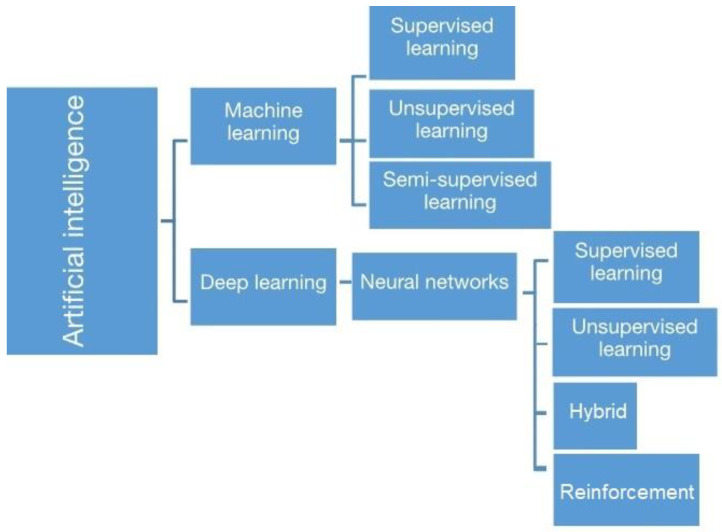
Basic artificial intelligence subtypes based on the computer system learning process [9,10,11].

Artificial Intelligence (AI) is a part of computer science dedicated to the development of algorithms with the main objective of performing functions traditionally associated with human intelligence. For this purpose, the computer system acquires information from input or past data by means of the different subsets of AI, such as ML, neural networks (NN), and deep learning (DL) [6,7]. ML is an arm of AI that explores the construction of computational algorithms in order to build a computer system that learns from a predefined database. ML methods can be categorized according to the training data availability, the algorithm process, and the segmentation model applied; the three more used are supervised learning (where the data is pre labeled by the operator), unsupervised learning (where the data is unlabeled), and semi-supervised learning (where the data is labeled and unlabeled). As for deep learning (DL), it is a subfield of AI which is based on neural networks (NN). These artificial networks are composed of multiple interconnecting neuron layers. DL uses complex models that exceed the capabilities of machine learning tools such as logistic regression and support vector machines [8]. It is useful to review various types of learning tasks such as supervised, unsupervised, semi-supervised (hybrid), and reinforcement. The taxonomy of these tasks is based on how they are used to solve various problems (Figure 1) [9,10,11].

DL is a subset of AI that uses artificial NN consisting of input and output data [12]. In this context, in the field of digital pathology, another important method for image-based DL is image segmentation. Image segmentation relies upon the separation of multiple parts from an initial whole slide image (WSI) in order to obtain images or objects of interest and cluster them according to their optical properties [13]. The acquisition of WSI allows the analysis and amplification of high-resolution and high-quality images, facilitating the visualization of stained tissue slides and even the exchange of these cases between oral pathologists [14].

## 4. Digital Image Analysis

The image processing and analysis system commonly used is the so-called ImageJ 1.53t©, which is an open-access software designed for the study of multidimensional pictures and is also the inception of FIJI 1.53t©, another open-access software available in the market [15]. It is considered highly practical, reproducible, impartial, and efficient, more than anything enriched by the scientific community that shares the constant development of tools called plugins. A plugin is a small part of the software dedicated to a specific task, such as color deconvolution, cell counter, color segmentation, and watershed transform, among others. This tool is created to assist pathologists and clinical practitioners in the decision-making processes and to reduce diagnosis delay and prognosis errors with the aim of promoting patients’ health.

According to Alabi et al., ML applications in oral cancer cover a large panel of areas that range from clinical-pathological-genomic data combination to image and autofluorescence information; they determined that deep NN was one of the most widely used approaches for oral cancer analysis [16]. Deep NN is an AI model inspired by the human brain, processing information one layer at a time, extracting and labeling relevant data through the layers in order to classify new data [17]. Despite the precision and objectiveness of the models, the authors stated that diagnosis and prognosis are hard to achieve with few real contributions to the medical field. Algorithms are usually constructed for a specific type of tissue, making it difficult to generalize the procedures for other entities that do not share the same characteristics. Moreover, studies can be limited by the number of images available to process, making it challenging to obtain an adequate amount of training and testing datasets.

## 5. Whole Slide Images

**Figure 2:**
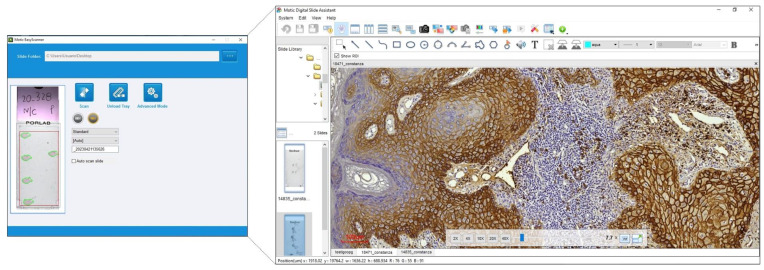
Overview of the scan of a histological slide and the result as a whole slide image (Motic EasyScan One Digital Slide Scanner, Motic Asia, China, Hong Kong) (Material extracted from the equipment of the Molecular Pathology Area, School of Dentistry, Universidad de la República, Montevideo, Uruguay).

In pathology, the use of WSI serves several benefits. WSI provides the opportunity to transform entire tissues on glass slides into digital high-resolution virtual slides [18]. Figure 2 shows an overview of a specific field of a WSI of OSCC. In this context, WSI appears as an important tool for the use of different AI algorithms for both diagnostic and research fields. This tool seems to have several advantages when compared to more conventional techniques such as optical microscopy since, in addition to the possibility of using AI, as already mentioned, the digitized file allows the maintenance of the quality of the staining techniques used in the tissue and it becomes portable and easy to share with other pathologists worldwide [19].

Nowadays, some studies have applied digital images and, more specifically, WSI for the use of AI methods for different approaches in head and neck squamous cell carcinoma (HNSCC). These methodologies have the advantage of automated quantification in WSIs of tissue slides, which enables the development of more robust analyzes of results, reducing the bias of the operators. In the study of Sung et al., it was used immunohistochemistry, WSI, and semiautomated tools in order to propose a scoring system for predicting pathological risk in OSCC [20]. Regardless of the specific results of the study, the authors demonstrated the use of a designed novel method that made it possible through WSI and image findings for the quantification and automated measurement of tumor area, tumor-infiltrating lymphocytes, and tumor budding. In another study, it was demonstrated the development of an automated score for the quantification of tumor-associated stroma infiltrating lymphocytes in WSIs [21]. Based on the advantages of these modern techniques, the methodology allowed the quantification of lymphocytes in the adjacent areas of tumor-associated stroma using the spatial co-occurrence statistics of both tumor-associated stroma and lymphocytes. This study showed for the first time an automated quantitative score of lymphocytic infiltration in the tumor-associated stroma of HNSCC. These are some examples of novel studies that demonstrate the objectivity and reproducibility advantages of automated quantification through WSIs.

WSIs have also served as a tool to investigate parameters with diagnostic purposes in OSCC; for example, another study’s authors digitized WSIs of 90 cases of OSCCs to develop an AI training method that can recognize both cellular and structural atypia in this type of tumor [22]. From that, the authors used a convolutional neural network (CNN) model to train and evaluate 90.059 image patches of OSCC with different sizes and showed that the method focused on both cellular and structural atypia, concluding that AI could be trained in order to evaluate these parameters and may be suitable for diagnosis of OSCC. In this context, Halicek et al. used 192 digitized WSIs to investigate the ability to detect squamous cell carcinoma [23]. The digitized histological images from patients with HNSCC were used to train, validate, and test a CNN, and the study also showed the potential of WSI to increase both efficiency and accuracy of pathologists in the detection of squamous cell carcinoma. As described, several advantages are related to advances in digital pathology and, in particular, to the use of WSIs. With digitized files, pathologists around the world can work with digital capture, storage, sharing, visualization, and with the use of different advanced techniques for numerous analyzes through specific software. Moreover, conservation over time and storage capacity are also benefits that this kind of technology has and should be considered a short-term implementation through pathology centers.

## 6. Image Segmentation

Automatic segmentation of digitized histological images in regions that represent different types of tissues is of high importance in developing digital diagnosis, prognosis, and therapeutic tools. The segmentation technique is a computational procedure that processes digital images by grouping pixels with similar colorimetric properties in regions that probably represent objects of interest (for example, cells, vessels, and other structures in the tissue). These can then be characterized geometrically in order to obtain qualitative and quantitative information about the objects that they represent [24,25]. The most commonly used soft-wares for applying this technique are ImageJ and FIJI, as we mentioned before, which are constantly developing new plugins and tools in order to achieve specific objectives; thresholding, StarDist, watershed transform, trainable WEKA segmentation, Labkit, among others, are some of the ones used, and also explained below, for image segmentation.

Pattern recognition techniques are other types of segmentation methods in which certain characteristics are selected, such as color, shape, and size, and afterward, results are clustered in regions that can correspond to histological classes [24,25]. Most of the methods applied to histopathological images stained with H&E, immunohistochemistry, and histochemical procedures are based on thresholding procedures, starting from a threshold value of color or intensity where the object of interest is identified [26]. In this manner, thresholding is a tool that clusters pixels based on shared characteristics. The result is a binary image with a value of 1 or 0 for the object of interest and the background. This procedure is part of a complex process that allows pathologists to determine the presence or absence of certain components in tissue samples, such as in the steps used in Pereira–Prado V et al. [27] for comparing odontogenic entities.

**Figure 3:**
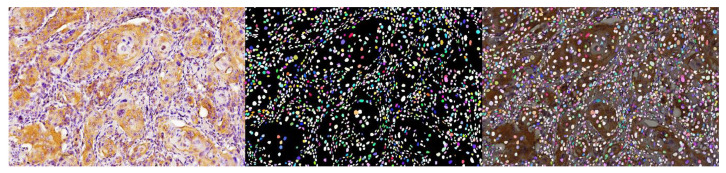
StarDist 2D segmentation in an OSCC image stained with immunohistochemistry. Note the precise nuclei identification and the superposition of both images.

Another relatively recent segmentation technique based on DL and NN is StarDist (Germany, 2018). It consists of the detection of a cell nucleus predicting its morphological profile, and being flexible and precise enough to compete with other segmentation methods. StarDist is based on a star-convex-polygon shape to approximately represent the round nuclear morphology [28]. This novel model allows for processing images not only for histological H&E format but also for fluorescent nuclei detection. Obtaining nuclear definition makes it possible to also establish several morphological nuclear parameters as well as to study nuclear density and condensation (Figure 3).

Watershed transform is a morphological segmentation method that splits objects of interest with watershed lines in catchment basins, constructing three-dimensional topographic maps (considering the intensity as the altitude of the sample) in which water immersion is simulated. In this manner, watershed lines are defined when different catchment basins contact each other. For histopathological purposes, this technique, using nuclear location and intensity, allows for the segmentation of tissues in virtual cells or v-cells to obtain morphological characteristics of cells and layers [29]. In order to apply the plugin, images must be in 8-bit binary type (0 and 255), and white background would be the one segmented.

**Figure 4:**
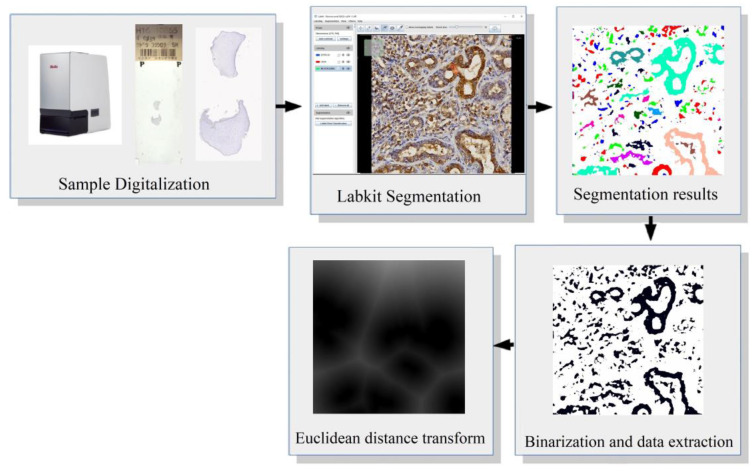
Labkit segmentation display with an OSCC image stained with immunohistochemistry. Note that the tool allows for selecting the number of classes to classify and segment the image. The segmentation results identify several tissue components that can be measured and compared.

Trainable WEKA (stands for Waikato Environment for Knowledge Analysis, New Zealand, 2009) Segmentation as well as Labkit (stands for Labeling and Segmentation Toolkit for Big Image Data, Germany, Figure 4) are two open softwares that combine FIJI, ML, and FF algorithms to segment and classify pixels [30,31]. In order to use these tools, the operator has to know and label which objects of interest want to be recognized by the software prior to its training. Labeled objects of interest are used as examples to train the model and classify new images [30,31]. In this manner, histological images can be segmented in order to determine structural components, from epithelial and connective layers to nuclei, basal membranes, cells, and vessels. Moreover, this kind of technique allows for the differentiation of images according to their staining, for example, segmenting objects stained with immunohistochemistry and the background with Mayer’s hematoxylin.

## 7. Comparing Data Results

One of the problems that AI procedures pertain to the comparison between samples. Pathologists should operate under the same protocol in order to compare their results: magnification, resolution, size of the image, and staining standardization procedures, among others. This can also fall into bias due to the use of the same dataset for model selection and evaluation of the model. To avoid bias, Mahmood et al. recommended the division of the dataset into three groups: model training, optimal model selection, and validation of the model, as well as adding new data to the last two groups [32]. Moreover, the authors suggested the inclusion of samples from different pathology centers in order to increase diversity and biological variations from different demographic locations. Further, the application of supervised training techniques requires the human annotation of parameters to train the segmentation model; multiple pathologists should perform this task to minimize subjectivity and reduce inter-pathologist variation. Pre-processing images when there is no possibility of standardization of the manipulation of the samples is also possible: adjusting contrast and reducing noise helps to delineate structures and differentiate tissues; using filters to achieve this is recommended, facilitating standardization of images and managing to compare them.

## 8. Advances in Oral Cancer Research

Diagnosis and prognosis determination using AI software must rely on the knowledge of cancerous tissues and the identification of specific parameters that make the difference between states. In this manner, several research papers on this topic have been made about specific characteristics of these entities: keratinization and keratin pearl areas [33], stage differentiation using linear layer NN classifier and hyperspectral imaging [34,35,36], cell nuclei segmentation [37], immunohistochemical biomarkers [38,39], textural–shape–color features [40], among others. In this section, some of the recent advances in the use of AI specific to OSCC are presented.

A study by Pratama et al. determined the possibility of classifying OSCC from different sites based on RNA sequencing data using CNN, resulting in a poorer performance when differentiating histopathological features [41]. In another study, Santer et al. exploited the classification of cervical lymph nodes in locally advanced OSCC with ML and DL, finding an accuracy of 86% for the training and testing sets [42]. Irrespective of the selected software, this suggests that quantitative AI analysis is a promising diagnostic support tool.

The research conducted by Das et al. differentiated OSCC from normal tissue using 1224 histopathological images of the oral cavity (290 normal tissue and 934 cancerous tissue) and applied CNN frames. As a result, this DL approach showed 82% accuracy compared to other state-of-the-art models, suggesting its application as an automated tool to identify oral cancer [43]. In another study, Yang et al. demonstrated that a developed custom-made DL model improved the accuracy and the speed of the diagnosis of OSCC [44]. Rahman et al. showed that a model of transfer learning using AlexNet in the CNN to predict oral cancer using OSCC biopsy images by means of performance parameters such as classification accuracy, classification miss rate, sensitivity, specificity, F1-score, positive predicted value, negative predicted value, false positive ratio, false negative ratio, likelihood positive ratio, likelihood negative ratio, and Fowlkes–Mallows index reached 90.06% of accuracy [45]. Therefore, the authors proposed that the studied model can be perfected by collaborations in the medical field.

In terms of the prediction of survival of oral cancer patients, Kim et al. compared in a retrospective study of 225 patients a DL-based survival prediction method (DeepSurv) with classical statistical methods [46]. By calculating Harrell’s c-index, it was demonstrated that DeepSurv presented the best performance among the compared methods. Similarly, Tseng et al. established through data mining a model for predicting a 5-year disease-free survival rate and a 5-year disease-specific survival rate of oral cancer patients using the traditional statistical method of logistic regression compared to the decision tree method and the artificial NN model [47]. The results showed a superiority of the decision tree and artificial NN when compared to the traditional method. Both studies demonstrate that AI may help in the prediction of the prognosis of oral cancer; however, more studies are necessary in this sense in order to support the provided information.

## 9. Role of AI in Immunofluorescence and Immunohistochemistry Oral Cancer Images

The application of immunohistochemistry and immunofluorescence for studying oral cancer behavior is widely known [48]. Biomarker expression analysis through quantitative digital techniques facilitates the interpretation of their possible implication, whether there is the expression or not in the pathological digital images. Kawamura et al. studied the expression of VEGF-C, VEGF-D, NRP1, NRP2, CCR7, and SEMA3E in 1854 images of 76 patients with OSCC using a multilayer perceptron NN [49]. Results determined an accuracy of the model of 98.6% to assess the staining levels (high or low) without considering morphological features and also associating the results with the presence of cervical lymph node metastasis. The authors suggested that this model identifies cervical lymph node metastasis from primary tongue tumors.

Multiplex immunofluorescence imaging to predict combined positive scores of certain markers (such as PD-L1) has been analyzed in head and neck squamous cell carcinoma with DL and ML techniques [50,51]. Manual scoring by a pathologist consists of the score of PD-L1 expression on at least 100 tumor cells, which is laborious, time-consuming, and subjective. The proposed AI approach increases the number of tumors analyzed by the period of time, as well as the likelihood of responsiveness to immunotherapies [50,51]. Tsakiroglou et al. also studied PD-L1 immunofluorescence staining, as well as other markers, in oropharyngeal squamous cell carcinomas using DL and CNN to pre-process images and then segment them with QuPath software. The authors determined a new tool to support diagnosis and therapies targeting the PD-1/ PD-L1 pathway of immune escape [42].

Table 1 shows a compilation of the research work applying AI for the study of oral cancer.

## 10. Discussion

Leaving aside clinical considerations that contribute to the differential diagnosis of oral cancer and that the actual gold standard for tumor identification is the biopsy’s histopathological analysis by a pathologist, it is important to highlight that AI holds great promise in this manner and is gaining weight in medical areas [52,53].

However, there are opposite sides of concern regarding the utility of AI in oral cancer. From the research that this review compiles, some authors state that due to the lack of algorithm generalization, sample manipulation, amount and quality of tissue, and tissue variable morphology between patients with the same diagnosis, it is hard to obtain a precise diagnosis only with AI [16,54,55,56].

As already mentioned, the use of AI methods in the oncology field still needs large-scale validation. To regulate the use of AI-driven models in clinical oncology, it is crucial to provide evidence showcasing the efficiency and security of these models through further clinical cancer research taking into account factors such as integrity, origin, retention, and distribution of data, the precision and reliability of selected models, ethical considerations and the inclusion of patients in predictive model usage, as well as the legal ramifications associated with the utilization of patient data [57]. According to Luchini et al., there is currently documented evidence of 71 AI-associated devices that have already obtained official approval from the Federal Drug Administration (FDA) [58], being cancer diagnostics the most important field. Indeed, for the regulation of the development of AI in the oncology field, a comprehensive and interdisciplinary approach in light of all the achievements attained in this field should be followed.

On the other hand, it has been studied the possibility of identifying cellular and structural atypia in OSCC suggesting that combining WSI with AI algorithms could allow the automatic evaluation of these parameters and may be suitable for its diagnosis [22,23]. ImageJ and FIJI software and the plugins mentioned previously, such as thresholding, StarDist, watershed transform, trainable WEKA segmentation, Labkit, have been widely used as free open tools for digital analysis, introducing new ways of developing AI for specific purposes [24,25,26,27,28,29,30,31].

From a biological standpoint, studying the characteristics and histopathological changes of potentially malignant disorders that can occur previous to an OSCC would allow an early diagnosis and prevent its development. Several studies have been carried out in this line of research, as well as in the identification of elementary oral lesions, applying different models of AI. Authors state that contributing to the diagnosis of high-risk oral cancer lesions could improve patient survival rates when being assisted with AI models [56,57,58,59,60].

Although there is no valid AI technique for the diagnosis of oral cancer, DL and ML methods would assist clinicians in the making of decisions with more objective information, improving patient management and treatment options, and closing the gap between patients in remote areas with less accessible medical assistance.

## 11. Conclusions

The present systematic review discussed some of the advancements in the use of different AI methods in OSCC. Although much progress has been made in image analysis processes that could help health professionals on improving prognoses and diagnoses or treatment election, it is still a wide area that needs further research, primarily to enhance the understanding of the different digital methods. Furthermore, further research on the topic can validate the different tools in artificial intelligence with the possibility of greater security for their future use in clinical practice.

## 12. Future Directions

For future research, there are several limitations to surpass in order to conduct more sustainable results. Scanning the slides should be the gold standard in order to obtain WSI, being necessary to have the correct equipment. Increasing the number of cases and images included in the studies is also an important matter, supporting the evidence with more precise and unbiased results. Due to the rarity of the lesions, pathology centers should aim to work together, establishing a standardized protocol to follow when obtaining quality digital images in order to validate the results. Moreover, to increase the value of AI in clinical practice, more research is required on the clinical validation of these tools.

## Figures and Tables

**Table 1:** Resume of AI applications mentioned in the present study.

Authors	Objective	Methods	Samples	Accuracy
Sung et al., 2021 [20]	Establish a scoring system for predicting pathological risk in OSCC	Immunohistochemistry, WSI, and semiautomated tools (in image J) for quantification of tumor area, tumor-infiltrating lymphocytes, and tumor budding	256 patients	Not determined
Shaban M et al., 2021 [21]	Establish an automated score for the quantification of tumor-associated stroma infiltrating lymphocytes	DL segmentation automated algorithm for the quantification of tumor-associated stroma infiltrating lymphocytes in WSIs	342 SCC cases from different sites of the head and neck, 100 OSCC, and 95 OPSCC.	85%
Oya K et al., 2022 [22]	Establish diagnostic parameters in OSCC	WSI. CNN using EfficientNet B0	90 cases of OSCC	99.65%
Halicek et al., 2019 [23]	Investigate the ability of AI to detect SCC	WSI, Aperio ImageScope, CNN	192 tissue specimens from 84 HNSCC patients	83.7%
Das et al., 2015 [33]	Identification of keratinization and keratin pearl areas in OSCC	Chan–Vese segmentation model	10 OSCC patients	95.08%
Prabhakar et al., 2017 [34]	Determine stage differentiation in oral cancer	Linear layer NN classifier	75 oral cancer patients	100% in T1, 85.19% in T2, 84.21% in T3 and 94.12% in T4
Jeyaraj et al., 2019 [35]	Determine a classification of oral cancer	DL CNN in hyperspectral imaging	1140 tumor sample information	94.5%
Halicek et al., 2017 [36]	Determine a classification of oral cancer	DL CNN using TensorFlow in hyperspectral imaging	DL CNN in hyperspectral imaging	80%
Mookiah et al., 2012 [37]	Determine a classification of oral submucosa fibrosis	Supervised and unsupervised cell nuclei segmentation	12 oral sub mucosa fibrosis patients and 10 normal tissue patients	Linear kernel based support vector machine 99.66%, Bayesian classifier 96.56% and Gaussian mixture model 90.37%.
Hu et al., 2014 [38]	Establish a rapid detection method for HNSCC	Linear-discriminant models using two or more measured optical biomarkers	57 patients	50–95%
Hameed et al., 2016 [39]	Determine an immunohistochemical scoring of oral cancer tissue images	ML classifiers such as support vector machine, k-nearest neighbor, linear discriminant analysis, and naive Bayes	12 OSCC samples	96.09%
Rahman et al., 2020 [40]	Evaluate malignancies in OSCC using digital imaging	WSI. Matlab. Classification methods: Decision tree, Support Vector Machine (SVM), Logistic Regression, Linear Discriminant and K-Nearest Neighbor	42 samples	99.4% Decision Tree Classifier, 100% SVM and Logistic regression, 100% SVM, Logistic regression and Linear Discriminant
Pratama et al., 2021 [41]	To aid diagnosis of OSCC	RNA sequencing data using ML and CNN	337 OSCC and other tissue samples	83%
Das et al., 2023 [43]	Aid the early detection of OSCC	DL, CNN	290 normal tissue and 934 cancerous tissue	82%
Yang et al., 2022 [44]	Assist pathologists in detecting OSCC from histopathology images	DL	2025 images	92%
Rahman et al., 2022 [45]	Predict oral cancer different parameters	A model of transfer learning using AlexNet, CNN	2511 OSCC images, 2435 healthy tissue images	90.06%
Kim et al., 2019 [46]	Prediction of survival of oral cancer patients	DL-based survival prediction method (DeepSurv)	225 patients	81%
Tseng et al., 2015 [47]	Prediction of 5-year disease-free survival rate and 5-year disease-specific survival rate of oral cancer patients	Data mining logistic regression compared to the decision tree method and the artificial NN model. WEKA.	673 cancer patients	63.3%
Kawamura et al., 2023 [48]	Predict lymph node metastasis in cancer by classifying the level of immunohistochemical markers	Multilayer perceptron NN	76 patients with OSCC	98.6%
Vahadane A et al., 2023 [49] Puladi B et al., 2021 [50]	Obtain reproducible and reliable scores of immunofluorescence imaging of PD-L1	WSI. DL and ML. QuPath. Matlab.	54 HNSCC	97.2%
Tsakiroglou et al., 2020 [51]	Quantify the frequencies of cell–cell spatial interactions occurring in the PD1/PD-L1 pathway	WSI. DL and CNN. QuPath.	72 OPSCC	88.3%

## Data Availability

No new data were created in the present study.
